# Successful Treatment of Olecranon Bursitis Caused by *Trueperella bernardiae*: Importance of Environmental Exposure and Pathogen Identification

**DOI:** 10.1155/2018/5353085

**Published:** 2018-09-04

**Authors:** Ivan Gowe, Christopher Parsons, Michael Best, Eveline Parsons, Scott Prechter, Stephen Vickery

**Affiliations:** ^1^Infection Preventionist, Margaret R. Pardee Memorial Hospital, 800 North Justice Street, Hendersonville, NC 28791, USA; ^2^Medical Director, Pardee Hospital Center for Infectious Diseases, 705 6th Avenue West, Suite D, Hendersonville, NC 28739, USA; ^3^Department of Microbiology, Margaret R. Pardee Memorial Hospital, 800 North Justice Street, Hendersonville, NC 28791, USA; ^4^Department of Animal Science, Berry College, P.O. Box 493259, Mount Berry, GA 30149, USA; ^5^Department of Radiology, Margaret R. Pardee Memorial Hospital, 800 North Justice Street, Hendersonville, NC 28791, USA; ^6^Assistant Professor, Wingate University School of Pharmacy, 805 6th Avenue West, Suite 200, Hendersonville, NC 28739, USA

## Abstract

A livestock farmer with a history of arthropathy presented with unilateral olecranon bursal swelling and tenderness. Multiple wound and intraoperative cultures revealed growth of *Trueperella bernardiae*, a Gram-positive coccobacillus, with symptom resolution following appropriate antimicrobial therapy. To our knowledge, this is the first case report of olecranon bursitis caused by this organism. Coryneform bacteria are often regarded as contaminants in clinical cultures, but advanced techniques for species identification may reveal expanded pathogenic potential of individual species like *T. bernardiae* and elucidate epidemiologic clues for osteoarticular infections in these cases.

## 1. Introduction

Olecranon bursitis is a relatively common osteoarticular infection in humans typically caused by *Staphylococcus* and *Streptococcus* species. Emerging data suggest that *Trueperella bernardiae*, a recently identified Gram-positive coccobacillus, is the cause of infections associated with surgical procedures and in the setting of comorbid conditions, including diabetes mellitus. We report the first case, to our knowledge, of olecranon bursitis caused by *T. bernardiae* and arising in a patient having significant contact with swine known to harbor this organism.

## 2. Case Report

**Figure 1:**
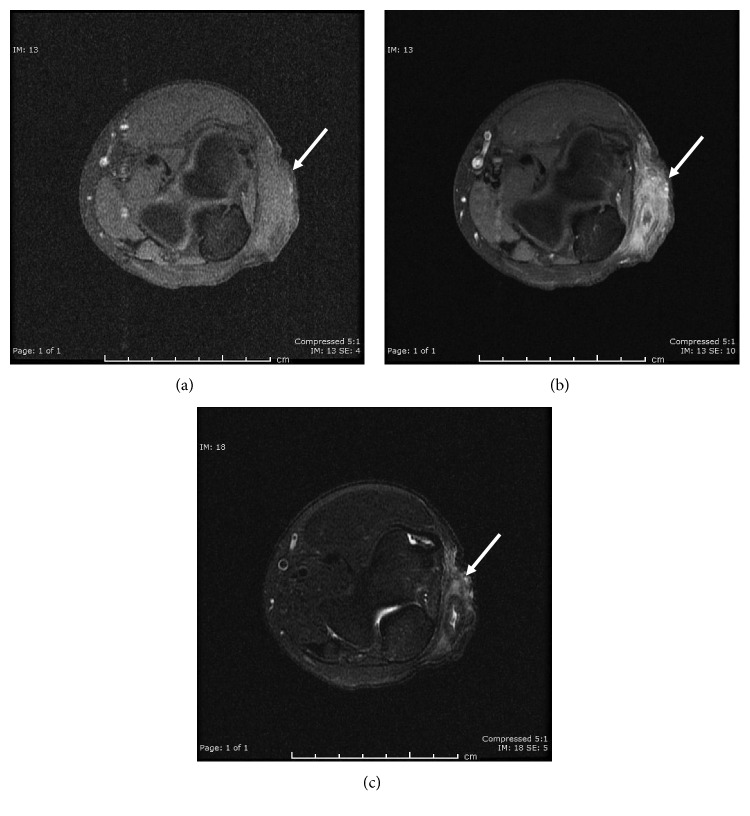
Pre- and postcontrast magnetic resonance imaging of right olecranon. (a) Axial T1 fat-saturated image demonstrates nonspecific T1 isointense mass-like enlargement of the subcutaneous soft tissues overlying the anconeus muscle along the posterolateral aspect of the elbow. (b) Axial T1 fat-saturated postcontrast image demonstrates central hypoenhancement correlating with T2 hyperintense phlegmonous collection (c). There is intense surrounding enhancement representing inflammation. (c) Axial T2 fat-saturated image demonstrates a 1.1 × 2.3 cm T2 centrally hyperintense, peripherally hypointense, phlegmonous collection with diffuse surrounding T2 hyperintense signal representing edema. The phlegmonous collection extended approximately 8 cm in largest dimension.

A 57-year-old male presented to an outpatient orthopedic clinic with a two-month history of pain and swelling in his right elbow. His past medical history was notable for hypertension and gout, which was controlled with colchicine, febuxostat, and indomethacin. He had undergone right olecranon bursectomy and tenotomy approximately two years prior to presentation, but reported good healing and no pain following this procedure, and otherwise denied recent trauma to the elbow. In addition, in his profession as a farmer, he routinely worked within a fenced area containing horses and pigs, including working on his elbows to clear brush and animal waste. On physical examination, he was noted to be febrile with an oral temperature of 41°C. A small aperture with purulent drainage was noted at the superior portion of his right olecranon bursa. Aspiration of bursal fluid was performed, with microscopic evaluation revealing rare polarizable crystals consistent with pseudogout. Following aspiration, the patient was admitted to the hospital. Initial laboratory data were notable for serum sodium 126 mmol/L, serum creatinine 178 *μ*mol/L (elevated from his baseline value of 75 *μ*mol/L), serum glucose 36.8 mmol/L, hemoglobin A1c 14.2%, white blood cells 7.4 × 10^9^/L, and erythrocyte sedimentation rate 14 mm/hr. Magnetic resonance imaging revealed a prominent soft tissue mass adjacent to the olecranon bursa and the posterior aspect of the medial epicondyle (Figure 1). The patient underwent incision and debridement of the right olecranon bursa, and intraoperative cultures were obtained. After this procedure, infectious disease consultation was requested, and an antimicrobial regimen consisting of intravenous (IV) ceftriaxone and vancomycin was initiated. On postoperative day 1, cultures from the initial bursa aspiration in the outpatient setting revealed diphtheroids. Subsequent identification of *Corynebacterium jeikeium* was reported 24 hours later by the hospital-based Vitek 2® microbial identification system. On postoperative day 3, the patient was clinically improved, without significant pain, and was discharged from the hospital to receive oral doxycycline 100 mg twice daily for an additional 14 days. Due to concern for infection, as well as the patient's renal function, he received neither corticosteroids nor nonsteroidal anti-inflammatory medication during or following hospitalization. Both aspiration and intraoperative cultures were sent to a reference laboratory (Mayo Medical Laboratories, Rochester, MN, USA) for bacterial identification and in vitro susceptibility testing. *T. bernardiae* was isolated as the sole pathogen utilizing matrix-assisted laser desorption ionization-time of flight (MALDI-TOF) mass spectrometry (score 2.1 with homology of the top ten results). In vitro susceptibility was determined by use of manual agar dilution which demonstrated activity of doxycycline as well as other antimicrobial agents (Table 1). Approximately two weeks following discharge, the patient was evaluated in the outpatient infectious disease clinic with marked improvement and no significant edema, erythema, pain, or drainage from his right elbow.

## 3. Discussion

Inflammation of the olecranon bursa (fluid-filled sacs that support musculoskeletal movements) may arise from septic and nonseptic causes [1]. Septic bursitis results from either bacterial introduction into the bursa or translocation from a surrounding skin-skin structure infection [2]. Olecranon or patellar bursae are commonly involved [3]. Gram-positive organisms are predominantly implicated, including *Staphylococcus aureus* and beta-hemolytic *Streptococcus* species [4]. Empiric treatment typically consists of a first-generation cephalosporin, vancomycin, or combination therapy depending on risk for MRSA infection or epidemiologic data potentially suggestive of infection with Gram-negative bacteria.

Septic olecranon bursitis should be suspected in a patient with elbow pain, redness, and swelling in the absence of pain aggravated by flexion and extension of the associated joint. Physical examination is typically notable for fever, peribursal cellulitis, bursal warmth, and tenderness [1, 2]. Diagnosis is confirmed with aspiration of bursal fluid demonstrating elevated white blood cell (WBC) count (typically >10,000/mm^3^), neutrophil predominance, and positive Gram stain [5].

Analysis of aspirated bursa fluid in this case also revealed crystals consistent with pseudogout, and there are rare reports of mass-like presentations with gout and pseudogout [6, 7]. Whether *Trueperella bernardiae* may preferentially infect joints or bursae affected by crystalline arthropathies is not clear, but this patient's clinical symptoms and signs improved dramatically with operative debridement and appropriate antibiotic therapy without nonsteroidal anti-inflammatory or corticosteroid therapy, supporting the pathogenic role of *Trueperella bernardiae* in this case.

*T. bernardiae* is a nonspore-forming, facultatively anaerobic, catalase-negative, Gram-positive coccobacillus. It is an uncommonly encountered commensal microorganism formerly categorized as group 2 coryneform bacteria [8–10]. After reclassification to the genus *Arcanobacterium*, this organism is now recognized as a member of the recently designated genus *Trueperella* [11]. Coryneform bacteria have been considered organisms of low pathogenicity, and their presence in human cultures is often interpreted as contaminants. However, recognition of the pathogenic potential of organisms within this class is vital, as increasing data have elucidated their role in infectious processes. Species identification of coryneform bacteria and susceptibility testing requires more sophisticated approaches. The organisms do not grow well on plated media leading to false-negative culture results [12–14], and *Trueperella* species are often confused with coryneform bacteria using biochemical methods alone [12]. In this case, the Vitek 2 identified the organism as *Corynebacterium jeikeium*, consistent with published data indicating a relatively significant degree of disagreement between MALDI-TOF and API (Remel) identification [13]. In addition, recent taxonomic changes have led to potential discrepancies in species identification if software or hard copy reference literature are not updated. This case report illustrates the superiority of MALDI technology in identifying these species.

Species identification of coryneform bacteria will also help establish epidemiologic connections for these infections. Following the recognition of *T. bernardiae* as pathogenic in humans, the strain was identified for the first time within stool collected from a newborn piglet, displaying both biochemical and mass spectrometry markers virtually identical to strains of *T. bernardiae* isolated in the context of human skin and soft tissue infections [15–17]. This is consistent with our patient's report of working directly with pigs, clearing their excrement and with his elbows positioned on the floor of the enclosed area. A related species, *Trueperella pyogenes*, is recognized as a cause of infections in bovine and porcine hosts, including mastitis, abscesses, and pneumonia [18], and characterization of *T. bernardiae* infection of animal hosts is ongoing. Regardless, these data support the possibility of human *T. bernardiae* infection resulting from exposure to porcine stool or other forms of contact with domestic animals as with our patient.

Optimal pharmacotherapy for *T. bernardiae* infections has yet to be elucidated. In vitro susceptibility to beta-lactams, clindamycin, tetracycline, and vancomycin is frequently observed; resistance to ciprofloxacin, aminoglycosides, and metronidazole has been described [19–21]. Given the paucity of susceptibility data, minimum inhibitory concentration interpretive criteria for the genus *Corynebacterium* can be extrapolated to *Arcanobacterium* (*Trueperella*) species for interpretation [22]. Effective treatment of osteoarticular infections caused by *Trueperella* species has consisted of fluoroquinolones [20, 23] and clindamycin [9, 21] for durations of 2 to 12 weeks. Our patient was successfully treated with a two-week course of doxycycline which, to our knowledge, has also not been reported in the medical literature. Doxycycline was chosen for definitive therapy based on its activity against *Corynebacterium* species [24] and utility in osteoarticular infections [25, 26].

In summary, we report a case of olecranon bursitis caused by *T. bernardiae* in a diabetic patient with pseudogout. *T. bernardiae* has been reported in osteoarticular infections, including prosthetic joint infections [20, 23], septic arthritis [21], and osteitis [9]. However, to our knowledge, this is the first reported case of olecranon bursitis caused by this organism. Empiric treatment of septic bursitis with a first-generation cephalosporin or vancomycin might typically provide appropriate antimicrobial coverage for this organism, which was mistakenly identified as *C. jeikeium* using traditional biochemical methodology. This case highlights the value of MALDI systems in microbial identification and in expanding our understanding of the pathogenic potential of organisms usually considered contaminants.

## Figures and Tables

**Table 1:** Antimicrobial susceptibility of *Trueperella bernardiae* isolates.

Antibiotic	MIC (mcg/mL)	Interpretation
Penicillin	≤0.06^a^, 0.12^b^	S
Ceftriaxone	≤0.5	S
Meropenem	≤0.25	S
Vancomycin	≤1	S
Doxycycline	0.5	S

^a^Aspiration culture results; ^b^operative culture results.
